# EMC2 promotes breast cancer progression and enhances sensitivity to PDK1/AKT inhibition by deubiquitinating ENO1

**DOI:** 10.7150/ijbs.109192

**Published:** 2025-03-24

**Authors:** Shihan Xiao, Shangxuan Jiang, Chengxu Wen, Han Wang, Wenxiang Nie, Jianguo Zhao, Bo Zhang

**Affiliations:** 1 Department of Breast and Thyroid Surgery, Union Hospital, Tongji Medical College, Huazhong University of Science and Technology, Wuhan, China.; 2 Department of Thyroid and Breast Surgery, Wuhan No. 1 Hospital, Wuhan, China.

**Keywords:** Breast cancer, EMC2, Scaffold, ENO1, Deubiquitination, PDK1, Drug sensitivity

## Abstract

Breast cancer is the most common malignant tumor worldwide, causing 685,000 deaths in 2020, and this number continues to rise. Identifying the molecular mechanisms driving breast cancer progression and potential therapeutic targets are currently urgent issues. Our previous work and bioinformatics analysis shows that the expression of Endoplasmic Reticulum Membrane Protein Complex Subunit 2 (EMC2) is up-regulated in breast cancer and is correlated with shortened overall survival of patients. However, the mechanism of EMC2 in breast cancer is yet to be elucidated. In this study, we identified that EMC2 promotes breast cancer proliferation and metastasis by activating the PDK1/AKT (T308)/mTOR (S2448) signaling pathway and can serve as a candidate target for PDK1/AKT inhibition. Mechanistically, EMC2 serves as a "scaffold" protein to recruit the deubiquitinating enzyme (DUB) USP7 for ENO1 deubiquitylation to stabilize its expression, thereby initiating downstream B-MYB/PDK1/AKT (T308)/mTOR (S2448) signal cascade. Silencing EMC2 significantly weaken the proliferation/metastasis potential of breast cancer *in vitro* and *in vivo*, but made tumor cell sensitive to PDK1/AKT inhibition. Overexpression of EMC2 leads to exactly the opposite result. This study reveals the EMC2/USP7/ENO1/B-MYB protumorigenic axis in breast cancer and identifies EMC2 as a candidate target for PDK1/AKT inhibitory therapy.

## Introduction

Breast cancer is the most prevalent malignant tumor among women. According to data published by Lancet (2024), breast cancer accounted for approximately 685,000 deaths globally in 2020 1. Currently, chemotherapy remains the cornerstone of breast cancer treatment, to which endocrine therapy or targeted therapy is added depending on hormone receptor (HR) expression 2. However, breast cancer is a highly heterogeneous disease. Even within the same subtype, there are significant differences in tumor growth rate, metastasis patterns, and chemotherapy sensitivity 3,4. As a result, some viewpoints also hold that the HR status cannot fully reflect the biological characteristics of tumors 5. This also results in some patients being unable or less likely to benefit from the treatment 6. Therefore, clarifying the molecular mechanisms driving breast cancer progression and identifying novel biomarkers with the potential to guide drug therapy are key scientific issues that need to be addressed.

The endoplasmic reticulum membrane complex (EMC) is an important complex of nine protein subunits on the endoplasmic reticulum of eukaryotic organisms 7, that is involved in the synthesis, folding, modification and processing of membrane proteins. EMC usually acts as a membrane protein insertion enzyme, recognizing and inserting newly synthesized tail-anchored protein (TA protein). For example, it utilizes its own transmembrane domains and hydrophilic "client" protein pockets to accommodate and stabilize the transmembrane domains of the TA protein to complete the insertion process 8. The rate of TA protein synthesis and membrane localization can be influenced by regulating EMC activity or expression levels 9. Specific subunits of the EMC complex (e.g., EMC3) are also involved in specific biological processes, such as vascular development 10, by modulating the correct folding and expression of receptors of signaling pathways (e.g., FZD4). The EMC is also capable of guiding the hydrophobic transmembrane helices of transmembrane domain (TMD) proteins to insert correctly into the lipid bilayer and is involved in the folding and assembly process of TMD proteins 7.

Many studies have revealed that EMCs mediate a variety of biological processes through different mechanisms. For example, EMC10 regulates hepatic endoplasmic reticulum stress and steatosis in an isoform-specific manner. The secreted isoform (scEMC10) promotes the activation of PERK-eIF2α-ATF4 signaling in hepatocytes, whereas the membrane-bound isoform (mEMC10) inhibits these signals 11. In addition, EMC-mediated biogenesis of the nonstructural multipass transmembrane proteins NS4A and NS4B has been suggested to be critical for dengue and Zika virus infections 12.

To date, preliminary studies have revealed the relationships between some subunits of EMC and cancer. For example, EMC3 is able to modulate the aberrant transport of the lung surface-active protein C mutant SP-C (I73T) and the associated cytotoxic damage 13. In lung adenocarcinoma, EMC6 is also involved in the regulation of immune cell infiltration, ferroptosis and cuproptosis 14. Overall, these sporadic clues are still insufficient to fully explain the mechanism of EMC in malignant tumors, especially breast cancer.

Endoplasmic reticulum-associated protein degradation (ERAD) is an intracellular protein quality control mechanism through which redundant proteins are ubiquitinated-proteasomal degradation after retranslocation from the endoplasmic reticulum to the cytoplasm 15. Recent studies have shown a close relationship between EMC and the ERAD pathway. On the one hand, large-scale mass spectrometry has revealed that there is significant crosstalk between EMC and ERAD in mammals 16. On the other hand, previous studies have shown that EMC and the ubiquitin-like chaperone UBQLN have a synergistic effect during the insertion and accumulation of the TA protein 8.

Here, we found that EMC2 expression is upregulated in breast cancer and is associated with poor overall survival. Silencing EMC2 significantly inhibited the proliferative and metastatic potential of breast cancer cells *in vitro* and *in vivo*. Moreover, drug sensitivity assays revealed that high EMC2 expression sensitized tumor cells to PDK1/AKT inhibition. Mechanistically, EMC2 stabilizes the expression of the RNA-binding protein ENO1 by acting as a 'scaffold' to recruit deubiquitinating enzyme-7 (USP7) to deubiquitinate ENO1. ENO1 subsequently promotes breast cancer progression by stabilizing the expression of the downstream transcription factor B-MYB, which ultimately activates the PDK1/AKT/mTOR signaling pathway thereby promoting breast cancer progression.

In summary, our study revealed the mechanism by which EMC2 promotes breast cancer progression and identified EMC2 as a drug target for PDK1/AKT inhibition.

## Methods

### Cell culture

All the cell lines used in this study were purchased from the American Type Culture Collection (ATCC). All the cell lines were authenticated via short tandem repeat (STR) profiling and confirmed to be mycoplasma-free. MDA-MB-231 and MCF-7 cells were grown in high-glucose DMEM supplemented with 10% fetal bovine serum. SK-BR-3 cells were grown in MyCoy's 5A medium supplemented with 10% fetal bovine serum. Penicillin-streptomycin solution at 1% was added to all media to prevent bacterial contamination.

### Animal experiments

The 5-week-old female BALB/c nude mice used to construct the subcutaneous graft tumor model and lung metastases were purchased from Charles River (Beijing, China). For the subcutaneous xenograft tumor model, 1×10^6^ tumor cells were transplanted into the right abdomen via subcutaneous injection, the tumor size was recorded at 3-day intervals, and the mice were humanely euthanized 35 days post inoculation or when the tumor weight reached 15% of the mouse's body weight. The volume of the graft tumor was calculated as follows: Volume (mm^3^) = Length * Width * Depth * (π/6). For the lung metastasis model, 1×10^6^ tumor cells (cells suspended in 200 µl of sterile saline) were injected through the tail vein, and the mice were sacrificed 7 weeks after inoculation. The lungs were weighed, and the number of tumor nodules was assessed.

### Drugs For treatment

For *in vivo* experiments, Capivasertib was administered orally by gavage (100 mg/kg body weight, twice daily) 17, while BX-795 was given orally by gavage (25 mg/kg body weight, twice weekly) 18. The control group received DMSO without any drugs as a placebo via oral gavage. Treatment was initiated on the third day following tumor implantation.

For *in vitro* experiments, the concentration of Capivasertib in the culture medium was 10 μM 17, whereas BX-795 was used at a concentration of 3 μM 19. The control group was cultured in a medium containing 0.5‰ DMSO.

All the aforementioned drugs were purchased from MedChemExpress (MCE), dissolved in DMSO, and stored at -80°C.

### Gene Silencing/Overexpression

The overexpression plasmids, silencing plasmids and negative control plasmids for EMC2 and ENO1 were obtained from Genechem (Shanghai, China). Small interfering RNAs for USP7 and B-MYB were provided by Sangon Biotech (Shanghai, China). Puromycin (working concentration: 1 µg/mL; Beyotime, China) was used to select stably silenced or stably overexpressing cell lines. The transfection of plasmids or siRNAs was performed in strict accordance with the instructions provided by the manufacturer (Lipo3000; Invitrogen, USA). Transfection efficiency was verified via quantitative real-time reverse transcription PCR (qRT‒PCR) and Western blot analysis. The shRNA or siRNA sequences of all the genes are available in Supplementary Table 1.

### Total RNA extraction and qRT‒PCR

Total cellular RNA was extracted via RNAiso Plus (TAKARA, Dalian, China). First-strand cDNA was synthesized via the HiScript®III Reverse Transcription Kit (Vazyme, China). qRT‒PCR was performed via a ChamQ SYBR kit (Vazyme, China). All steps were performed in strict accordance with the instructions provided by the manufacturer. For the calculation of relative RNA expression, the 2-ΔΔCT method was used, and the human GAPDH gene was used as an internal reference. All forward/reverse primer sequences are available in Supplementary Table 2.

### Western blotting

The cell lysis step was carried out in a refrigerator at 4°C. RIPA buffer (Biosharp, China) was supplemented with protease inhibitor cocktail (working concentration: 1%; MedChemExpress, USA) and phosphatase inhibitor cocktail (working concentration: 2%; MedChemExpress, USA) was used to prevent total or phosphorylated protein degradation. The lysate was broken by ultrasonic waves, and a 1/4 volume of 5X loading buffer (Beyotime, China) was added and mixed, followed by heating in a constant metal bath at 95°C for 5 min to fully denature the proteins. Protein electrophoresis was performed via 7.5-15% SDS‒PAGE, and the proteins were subsequently transferred to a nitrocellulose membrane (NC membrane, PALL, USA). Five percent skim milk was used to block the antigens at room temperature for 2 h, after which antibody hybridization detection equipment was obtained from Bio-Rad (USA). All the antibody sources and working concentrations used in this step are available in Supplementary Table 3.

### Immunocytochemistry (ICC)

The cells were cultured on glass coverslips and after 24 h, they were treated with 4% PFA fixative, 3‰ Triton, and 5% bovine serum albumin in sequence. This was followed by overnight incubation with primary antibody at 4°C.This was followed by incubation with secondary antibody cross-linked with green or orange fluorescent dye for 1 h at room temperature in the dark. A fluorescence inverted microscope was used for observation and imaging.

### Immunohistochemistry (IHC)

Immunohistochemistry was performed as previously described. In brief, the tissue sections were deparaffinized and sequentially subjected to antigen repair, 3% hydrogen peroxide treatment, antigen blocking, primary antibody incubation (4°C, overnight), secondary antibody incubation (RT, 1 h), DAB staining, and HE staining followed by observation under an inverted microscope and image capture. The level of staining was independently assessed by two experienced pathologists. The staining intensity scores were as follows: 0 (none), 1 (weak), 2 (moderate), and 3 (strong); the percentage of positive cells was 1 (≤10%), 2 (11-40%), 3 (41-70%), 4 (>70%). The final immunohistochemical score was as follows: staining intensity × percentage of positive cells.

### Cell proliferation assay

For the CCK-8 proliferation assays, 1000-3000 cells/well were grown in 96-well cell culture plates, and the absorbance (OD450) was determined by adding Cell Counting Kit-8 (CCK-8; MedChemExpress, USA) working solution every 24 h after inoculation. For the colony formation experiments, 500 cells/well were grown in 6-well cell culture plates, the medium was changed at 3-day intervals, and 0.5% crystal violet staining was used on day 15. The number of clones was automatically identified and counted via ImageJ software (version 1.8.0).

For the EdU incorporation assay, staining was performed according to the manufacturer's instructions (EdU488/555; Beyotime, China), and positive cells were detected via inverted fluorescence microscopy. The number of EdU-positive cells was automatically identified and counted via ImageJ software (version 1.8.0).

### Cell migration and invasion assays

For the wound healing assay, when the cells reached 95-100% confluence within a 6-well cell culture plate, a vertical scratch was created using 200 µL of peptide, and the culture was continued in medium containing 1% FBS. Images were taken at 0 h, 24 h and 48 h. The wound healing rate was calculated via the following formula: (initial width - width at the time of shooting)/initial width × 100%. For transwell migration experiments, 5×10^4^ cells or 1×10^5^ cells were seeded into the upper layer of transwell chambers (8 μm pore size; BD Falcon, USA) containing 200 µl of FBS-free medium, and the lower layer contained 600 µl of complete medium (10% FBS). After 24 hours of incubation, the cells were fixed and stained, and the lower layer was counted.

For the transwell invasion experiments, a matrix adhesive coating was precoated on the membrane on the basis of the transwell migration experiments.

### Bioinformatics analysis

Discrepancy analyses (DESeq package), survival analyses (Survminer package and survival package), and GSEA (Cluster Profiler package) analyses were performed with R software (version 4.4.2). Expression profiling data for breast cancer patients and corresponding clinical information were obtained from the public databases The Cancer Genome Atlas (TCGA, https://portal.gdc.cancer.gov/) and KM-plotter (https://kmplot.com/analysis/).

### IP, Co-IP and MS

For IP or co-IP, the cells were fully lysed via IP lysis buffer (Beyotime, China) containing a mixture of protease inhibitors, and the lysates were immunoprecipitated (4°C, overnight) with a target antibody or negative control antibody (IgG). Antibody‒protein complexes were captured via protein A/G magnetic beads (Beyotime, China) and subsequently detected via Western blotting or analyzed via MS. Mass spectrometry analysis, which is based on the quantitative method of unlabeled proteins (label-free), was subsequently performed at Genechem, Shanghai China.

### RNA-seq / RIP-seq & ChIP-qPCR

For RNA-seq, after total RNA extraction, RNA sequencing was performed by Majorbio Company (Shanghai, China). The ChIP assay was performed according to the instructions provided by the kit manufacturer (BeyoChIP™ Enzymatic ChIP Assay Kit, Beyotime, China), and for the final product, enrichment levels were detected via qRT‒PCR. The RIP assay was performed according to the instructions provided by the kit manufacturer (BeyoRIP™ RIP Assay Kit, Beyotime, China), and for the final product, the RIP assay was performed at Seqhealth Technology Ltd (Wuhan, China) for RIP-seq analysis.

### Cell viability assay

The half-maximal inhibitory concentration (IC50) was determined by the half-cell viability measured via an *in vitro* CCK-8 cytotoxicity assay, and the formula for cell viability was as follows: [(As-Ab)/(Ac-Ab)] × 100%; As: OD450 value of the treatment well; Ab: OD450 value of the blanked well; Ac: OD450 value of the control well. Each experimental group consisted of a DMSO control and a media only control. The maximum working concentration of DMSO was less than 0.1% 20.

### Statistical analysis

All experiments were conducted independently three times to determine the mean and standard deviation (s.d.). For continuous variables, analyses were conducted via Student's t tests, one-way ANOVA, or two-way ANOVA. For categorical variables, analyses were performed via Fisher's exact test. All the data were statistically analyzed via SPSS software (Version 22.0, IBM, USA) and R software (Version 4.2.0, USA). A p value < 0.05 was considered statistically significant. *: *P* <0.05; **: *P* <0.01; ***: *P* <0.001.

### Upregulation of EMC2 expression is associated with poor prognosis in breast cancer patients

**Figure 1:**
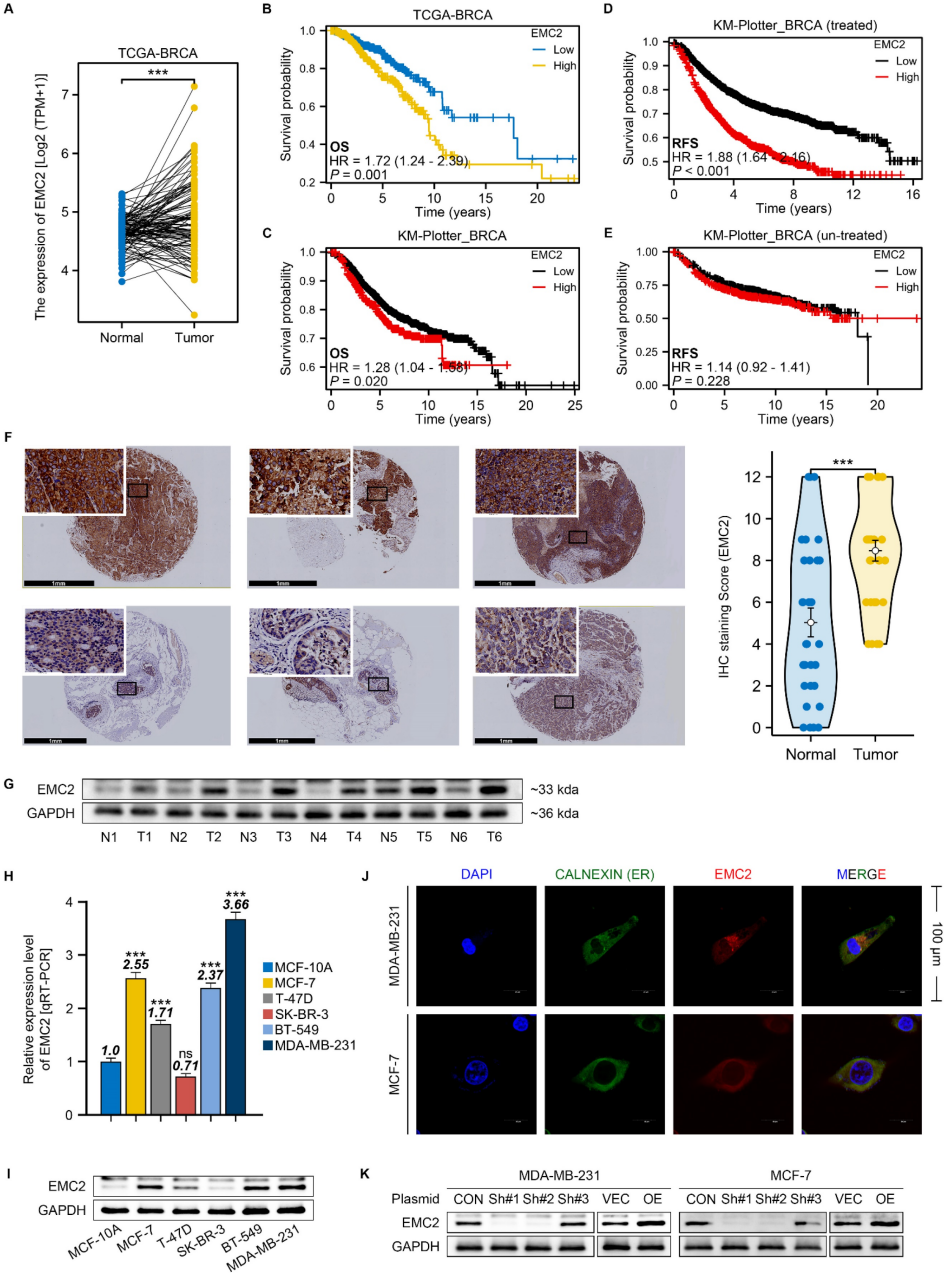
** EMC2 Expression Pattern and Clinical Significance in Breast Cancer. A.** Differential expression analysis of EMC2 using paired transcriptomic data from The Cancer Genome Atlas Breast Cancer (TCGA-BRCA) dataset. **B-C.** Kaplan-Meier analysis of overall survival (OS) based on EMC2 expression levels using transcriptomic and clinical data from TCGA and KM-plotter databases. **D-E.** Kaplan-Meier analysis of recurrence-free survival (RFS) based on EMC2 expression levels using transcriptomic and clinical data from TCGA and KM-plotter databases. **F.** Representative immunohistochemical staining and quantitative analysis of EMC2 expression in paired tumor and adjacent normal tissues. (Black scale bar: 1mm, and white scale bar: 100nm.) **G.** Western blot analysis of EMC2 protein expression in paired tumor and adjacent normal tissues. **H.** qRT-PCR analysis of EMC2 mRNA expression in one normal mammary epithelial cell line and five breast cancer cell lines. **I.** Western blot analysis of EMC2 protein expression in one normal mammary epithelial cell line and five breast cancer cell lines. **J.** Immunofluorescence co-staining of EMC2 and Calnexin in MDA-MB-231 and MCF-7 cells. **K.** Western blot validation of EMC2 protein expression following plasmid-mediated knockdown or overexpression.

Given the limited understanding of EMC2 expression patterns and mechanisms in breast cancer, we first searched for transcriptomic data and clinical information from the TCGA and GEO databases. Bioinformatics analysis revealed that EMC2 expression was upregulated in breast cancer (Fig. 1A) and was strongly associated with shorter overall survival (OS) in patients (Fig. 1B and C). Interestingly, we also found that patients with high EMC2 expression who received systemic therapy had significantly shorter recurrence-free survival (RFS) compared to those who did not receive systemic therapy (Fig. 1D and E). These findings suggest that the high expression of EMC2 in breast cancer may contribute to the poor prognosis of patients as well as drug resistance through several mechanisms.

We subsequently collected tissue samples from breast cancer patients at our clinical center and conducted immunohistochemical staining and Western blot analysis. The results clearly indicated that EMC2 is widely expressed in breast cancer tissues, with expression levels significantly higher than those in paired adjacent normal tissues (Fig. 1F and G).

We further examined several common human breast cancer cell lines to determine whether there are differences in the expression levels of EMC2 across different types of breast cancer. The qRT‒PCR and western blot results revealed that the expression levels of EMC2 were relatively higher in the MCF-7 (HR positive), MDA-MB-231 (HR negative) and BT-549 (HR negative) cell lines than in the MCF-10A (normal cell line), T47-D (HR positive), and SK-BR-3 (HER2 positive) cell lines (Fig. 1H and I). Immunocytofluorescence revealed that EMC2 was strongly expressed in MCF-7 and MDA-MB-231 cells and colocalized with ER biomarkers (Fig. 1J). Our results suggest that EMC2 is indeed upregulated in specific types of breast cancer cells and that this differential expression does not correlate with HR status. We then knocked down or overexpressed EMC2 in MCF-7 and MDA-MB-231 cell lines, respectively, for subsequent phenotypic and molecular studies (Fig. 1K, Fig. S1A and B).

### EMC2 promotes breast cancer proliferation *in vitro* and *in vivo*

**Figure 2:**
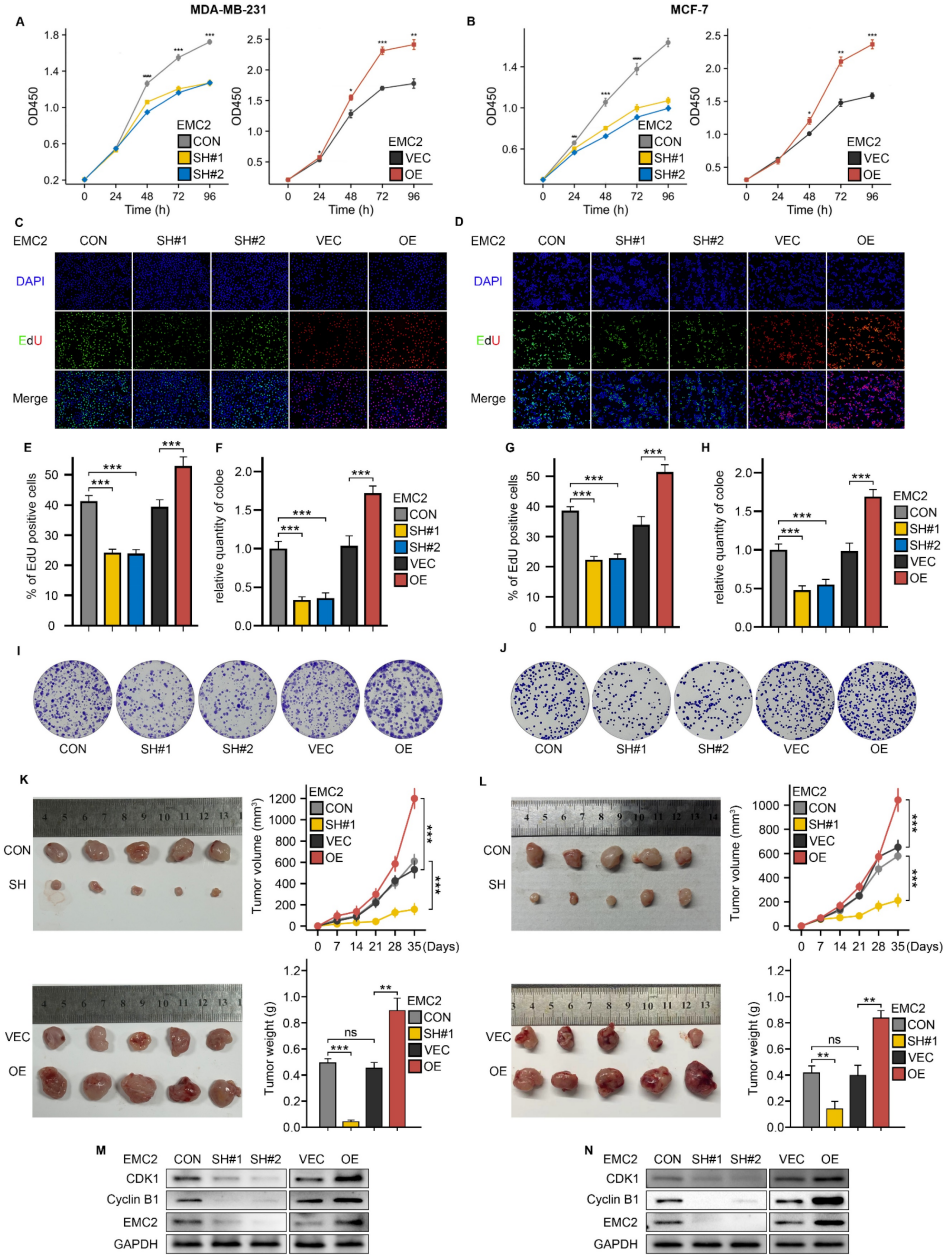
** Effects of EMC2 on Breast Cancer Cell Proliferation. A-B.** Cell proliferation analysis using CCK-8 assay in EMC2-modified MDA-MB-231 and MCF-7 cells. **C/D/E/G.** Cell cycle analysis using EdU incorporation assay in EMC2-modified cells. (Magnification 100X [10X objective lens × 10X eyepiece lens]) **F/H/I/J.** Colony formation assay in EMC2-modified cells. (6-well plate, diameter of 34.2mm) **K-L.** Xenograft tumor models were used to assess the effect of EMC2 silencing or overexpression on the proliferative capacity of MDA-MB-231 or MCF-7 cells. **M-N.** Western blot analysis of cell cycle checkpoint proteins.

Given that survival analysis revealed that upregulation of EMC2 expression was associated with shorter overall survival in breast cancer patients, we then performed CCK-8, EdU incorporation, and colony formation assays to detect cancer cell proliferation levels *in vitro*. The results revealed that EMC2 knockdown significantly decreased both the tumor cell proliferation rate (Fig. 2A and B) and the number of clones (Fig. 2F and H-J). In addition, the results of the EdU incorporation assay revealed that DNA synthesis was significantly blocked (Fig. 2C-E and G). The overexpression of EMC2 led to the opposite results. *In vivo*, EMC2 silencing significantly reduced the growth rate and final volume of xenograft subcutaneous tumors, whereas EMC2 overexpression accelerated the growth of transplanted tumors (Fig. 2K and L).

Finally, we examined the expression levels of several classical cell cycle-associated kinases and their ligands, and revealed that the expression levels of Cyclin-dependent kinase 1 (CDK1) and its typical ligand G2/mitotic-specific cyclin-B1 (Cyclin B1) which are responsible for regulating the cellular transition from the G1 phase (prephase of DNA replication) to the S phase (phase of DNA replication), were significantly and positively correlated with EMC2 (Fig. 2M and N, Fig. S2A and B).

These results consistently demonstrate that silencing EMC2 leads to cell cycle arrest (mainly during the DNA replication phase) and ultimately inhibits cell proliferation *in vitro* and *in vivo*.

### EMC2 promotes breast cancer metastasis *in vivo* and *in vitro* by mediating epithelial-mesenchymal transition

**Figure 3:**
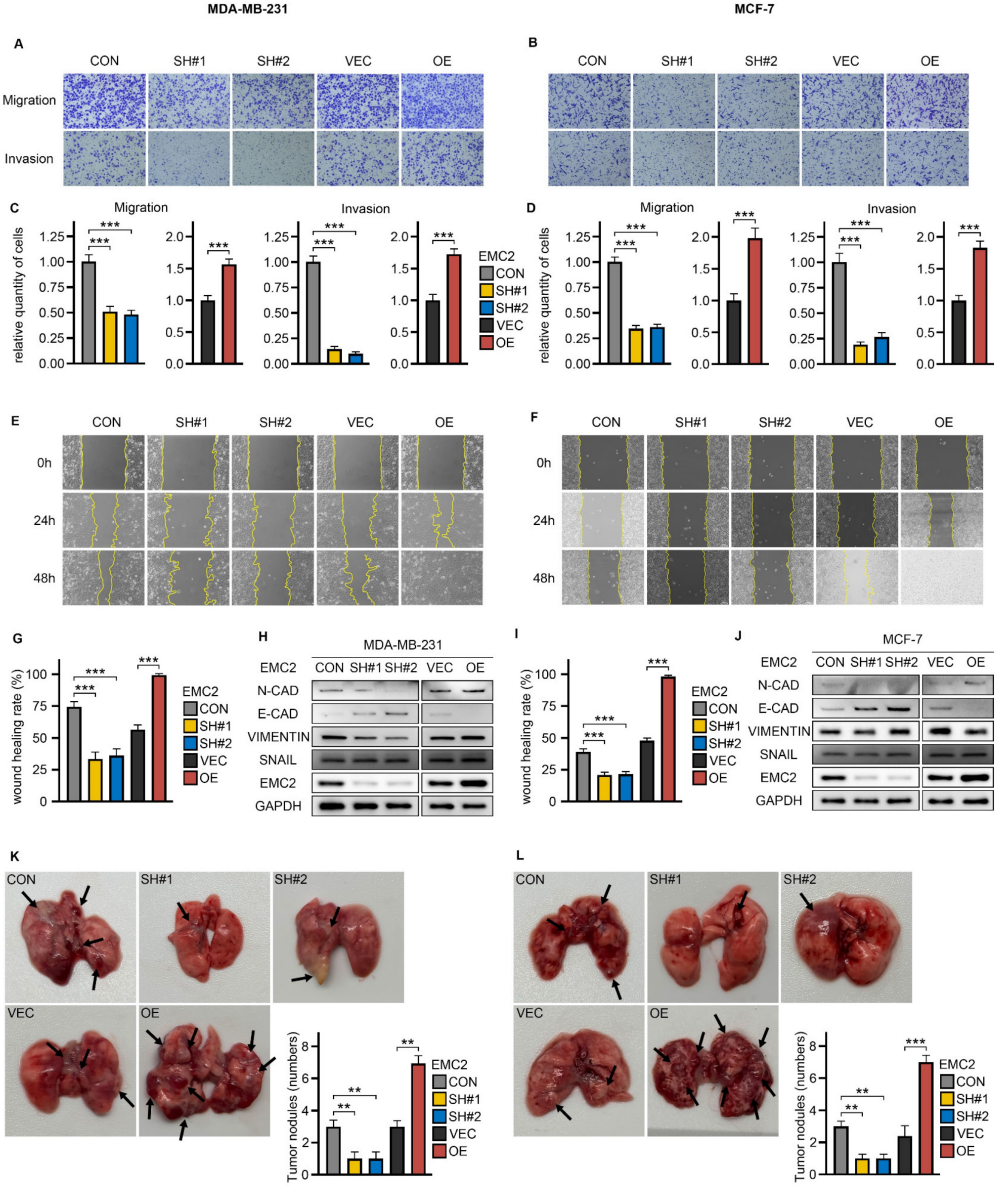
** Impact of EMC2 on Breast Cancer Cell Metastasis A-D.** Transwell migration and invasion assays in EMC2-modified cells. (Magnification 100X [10X objective lens × 10X eyepiece lens]) **E/F/G/I.** Wound healing assay in EMC2-modified cells. (Magnification 100X [10X objective lens × 10X eyepiece lens]) **H/J.** Western blot analysis of epithelial-mesenchymal transition (EMT) markers. **K-L.** Quantification of tumor nodules in xenograft metastasis model.

Breast cancer is a malignant tumor with a high propensity to metastasize, and we next investigated the effect of EMC2 on the metastatic potential of breast cancer cells. Through wound healing and transwell assays, we observed that the migration and invasion abilities of MCF-7 and MDA-MB-231 cells were significantly inhibited by silencing EMC2, which was mainly manifested as a significant decrease in the number of cells migrating to the lower chamber of the transwell (Fig. 3A-D) and the rate of wound healing (Fig. 3E-G and I). Breast cancer is a highly aggressive tumor of epithelial cell origin, and previous studies have established the important role of mesenchymal transition (EMT) in breast cancer metastasis. We detected significant changes in the expression levels of several EMT-related proteins with well-defined roles. In brief, N-cadherin (N-CAD) and vimentin (metastasis-promoting) were downregulated with EMC2 knockdown, whereas EMC2 overexpression increased their expression levels. In contrast, E-cadherin (E-CAD), which inhibits metastasis, was significantly negatively correlated with EMC2 expression levels (Fig. 3H and J).

Finally, in the xenograft lung metastasis model, EMC2 silencing led to fewer tumor nodules than in the control group (Fig. 3H and J). As predicted, the overexpression of EMC2 led to the opposite effect (Fig. 3K and L).

### EMC2 activates the PDK1/AKT/mTOR pathway by upregulating of ENO1

**Figure 4:**
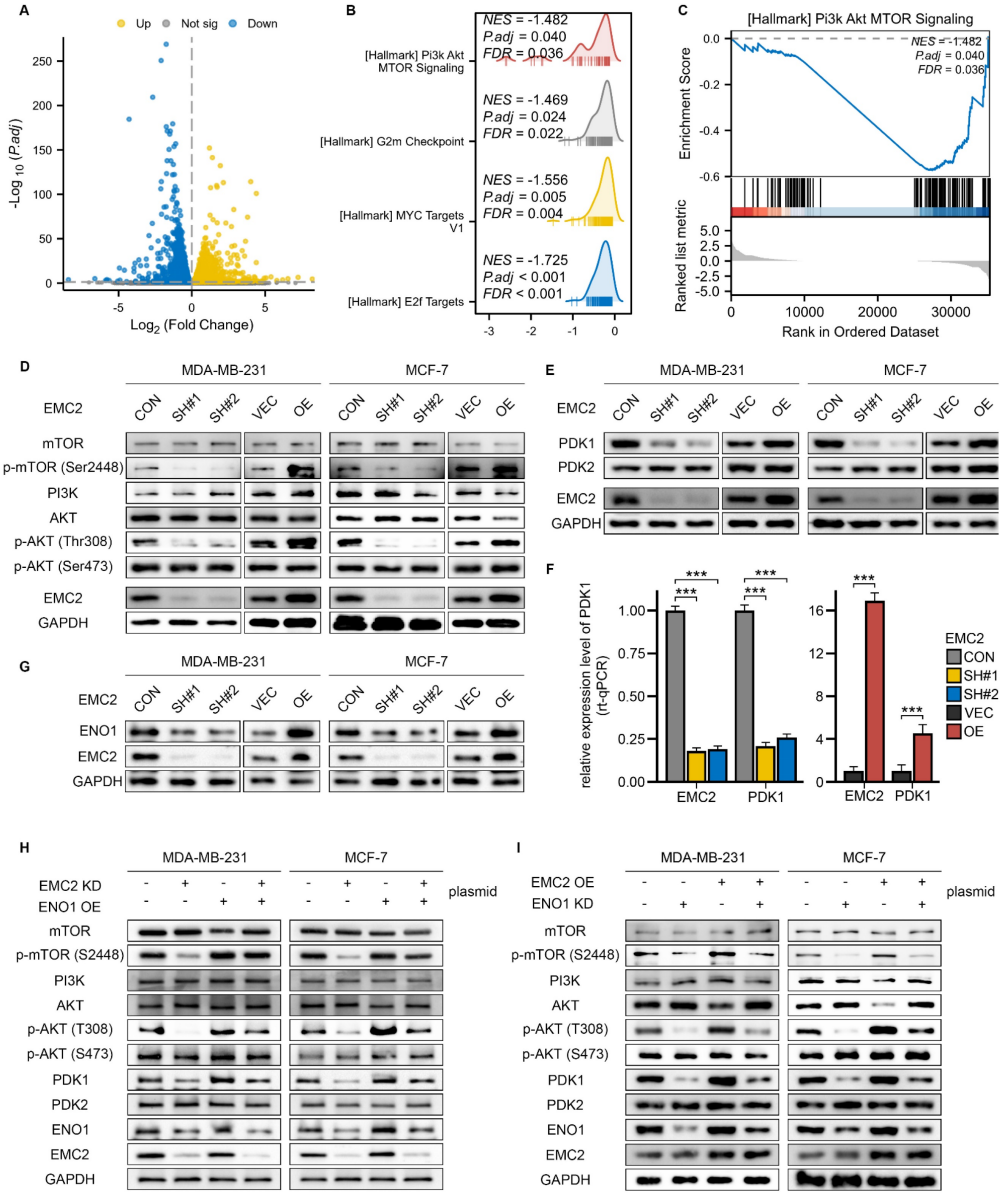
** Molecular Mechanism of EMC2 in PDK1/AKT/mTOR Pathway Regulation. A.** Volcano plot showing differential gene expression following EMC2 knockdown in MCF-7 cells. **B.** Gene Set Enrichment Analysis (GSEA) of signaling pathways following EMC2 knockdown. **C.** Butterfly plot demonstrating depletion of PI3K/AKT/mTOR pathway following EMC2 knockdown. **D.** Western blot analysis of PI3K/AKT/mTOR pathway components in relation to EMC2 expression. **E.** Western blot analysis of PDK1/PDK2 expression in relation to EMC2 levels. **F.** qRT-PCR analysis of PDK1 expression in relation to EMC2 levels. **G.** Western blot analysis of ENO1 expression in relation to EMC2 levels. **H-I.** Rescue experiments: Western blot analysis of PI3K/AKT/mTOR pathway components following: (H) ENO1 overexpression in EMC2-knockdown cells (I) ENO1 knockdown in EMC2-overexpressing cells

To investigate the mechanism through which EMC2 regulates the metastatic and proliferative potential of MDA-MB-231/MCF-7 cells, we subsequently determined the changes in the transcriptome landscape after EMC2 silencing via transcriptomics (RNA-seq). A volcano diagram indicated that silencing EMC2 resulted in changes in the expression levels of many genes (Fig. 4A). We then conducted gene set enrichment analysis (GSEA) to determine whether EMC2 silencing resulted in the enrichment or depletion of specific gene sets. Interestingly, the GSEA results revealed that four well-known pro-oncogenic gene sets were significantly depleted rather than enriched: phosphatidylinositol 3-kinase (PI3K)-AKT-mTOR/G2m checkpoint/MYC target V1/E2f (Fig. 4B).

We then focused on the PI3K-AKT-mTOR signaling pathway (Fig. 4C), as we noted that EMC6 (another member of the EMC family) mediated autophagy was associated with inactivation of the PI3K/AKT/mTOR signaling pathway 21. Many studies have demonstrated that the PI3K-AKT-mTOR signaling pathway is closely related to breast cancer development 22,23. The PI3K/AKT/mTOR signaling cascade is highly dependent on the phosphorylation activation mechanism; in short, in breast cancer, PI3K first phosphorylates and activates the Thr308 and Ser473 sites of protein kinase B (PKB, also known as AKT), after which AKT phosphorylates mammalian target of rapamycin (mTOR [Ser2448]) 24.

We first assessed the protein expression levels of members within the pathway and found that EMC2 silencing significantly inhibited the phosphorylation of AKT (Thr308) and downstream mTOR (Ser2448), with overexpression leading to the opposite result (Fig. 4D). Interestingly, we noted no significant changes in the expression levels of AKT (Ser473) or PI3K (Fig. 4D). These results suggest that EMC2 phosphorylates AKT(Thr308) via a PI3K-independent mechanism and ultimately activates mTOR (Ser2448). Pyruvate dehydrogenase kinase isozyme 1/2 (PDK1/2) are the key phosphorylated kinases of AKT. In brief, PDK1 and PKD2 are responsible for activating the Thr308/Ser473 phosphorylation sites of AKT, respectively. We then further examined the expression levels of PDK1/2, and the results revealed that PDK1 expression was significantly downregulated after EMC2 knockdown and that the overexpression of EMC2 further upregulated PDK1 expression, whereas PDK2 expression was not affected by the expression level of EMC2 (Fig. 4E). These results consistently demonstrated that EMC2 activated the AKT (Thr308)/mTOR (Ser2448) signaling pathway by increasing PDK1 expression.

We proceeded to consider whether PDK1 expression is regulated at the transcriptional level, and if so, by what mechanism. By qRT-PCR, we compared the expression levels of EMC2 and PDK1, and the results showed that in both MCF-7 and MDA-MB-231 cells, the mRNA expression level of PDK1 was positively correlated with EMC2 (Fig. 4F, Fig. S3A). Furthermore, the same trend was observed by transient silencing of EMC2 by small interfering RNAs Fig. S3B).

We further hypothesized that EMC2 performs transcriptional regulatory functions via protein‒protein coreactions and attempted to validate this hypothesis. In summary, we performed immunoprecipitation-mass spectrometry (IP‒MS) analysis of MCF-7 whole cell lysates. Interestingly, the IP‒MS results indicated that EMC2 directly or indirectly bound to a substantial number of client proteins (Table S4. After confirming that PDK1 is not a binding protein of EMC2, we focused on those proteins with relatively high binding abundance that have been reported to possess transcriptional regulatory functions.

Several recent studies have reported that enolase 1 (ENO1) functions as an RNA-binding protein to regulate target RNA transcript levels 25,26, and our mass spectrometry results revealed that the binding abundance of EMC2 to ENO1 was 2.5-fold greater than that of IgG controls Table S4. We next investigated whether EMC2 regulates the protein expression level of ENO1 by binding to it. Consistent with expectations, the protein expression level of ENO1 was clearly positively regulated by EMC2 (Fig. 4G), whereas at the transcriptional level, ENO1 mRNA was not regulated by EMC2 Fig. S3C).

To further determine whether the activation of the PDK1/AKT(Thr308)/mTOR(Ser2448) pathway by EMC2 upregulates ENO1 expression, we performed a series of rescue experiments via plasmid cotransfection. Encouragingly, the restoration of ENO1 expression in the EMC2 knockdown cell line effectively rescued the overall activation level of the PDK1/AKT(Thr308)/mTOR(Ser2448) pathway (Fig. 4H). Similarly, the inhibition of ENO1 expression partially abolished the pathway activation caused by the overexpression of EMC2 (Fig. 4I). Taken together, strong evidence suggests that EMC2 activates the PDK1/AKT(Thr308)/mTOR(Ser2448) signaling pathway by upregulating ENO1 expression and thereby activating the PDK1/AKT(Thr308)/mTOR(Ser2448) signaling pathway.

### EMC2 serves as a "scaffold" to recruit USP7 for deubiquitination of ENO1

We next investigated how EMC2 regulates the expression levels of the ENO1 protein. EMC2 is localized in the endoplasmic reticulum (27 and has been previously reported to be associated with the ERAD pathway 16 - a protein degradation mechanism based on the ubiquitin-proteasome system 28. We therefore attempted to identify potential EMC2-binding members of the ubiquitination system that might bind to EMC2. Encouragingly, we found that the binding abundance of ubiquitin specific peptidase 7 (USP7) to EMC2 was significantly increased (foldchange=1.35; Table S4).

**Figure 5:**
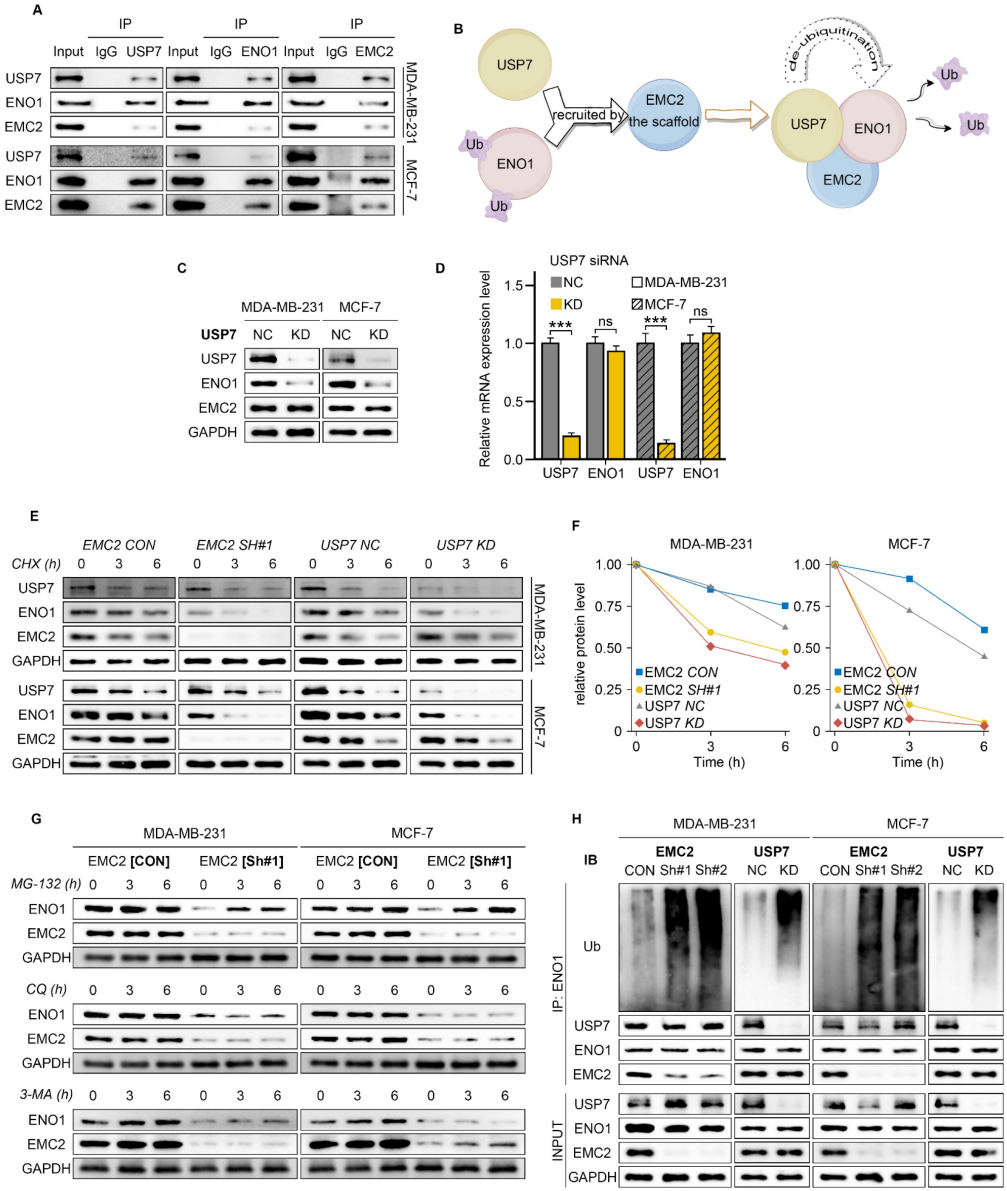
** EMC2-Mediated ENO1 Deubiquitination Mechanism. A.** Co-immunoprecipitation analysis demonstrating protein interactions between EMC2, ENO1, and USP7. **B.** Schematic hypothesis: EMC2 functions as a scaffold protein to recruit USP7 for the deubiquitination of ENO1. **C-D.** Effect of USP7 knockdown on ENO1 expression analyzed by Western blot and qRT-PCR. **E-F.** Protein stability analysis using cycloheximide chase assay (CHX, 50 μg/mL) at 0, 3, and 6 hours. **G.** ENO1 protein levels following treatment with protein degradation inhibitors: MG-132 (proteasome inhibitor, 10 μM), Chloroquine (CQ, lysosomal inhibitor, 50 μM), 3-Methyladenine (3-MA, autophagy inhibitor, 10 mM). **H.** Analysis of ENO1 ubiquitination levels following MG-132 (10 μM) treatment for 10 hours by Western blot.

Co-IP demonstrated that EMC2, ENO1 and USP7 bind to each other in the MDA-MB-231 and MCF-7 cell lines (Fig. 5A). We further hypothesized that EMC2 acts as a scaffold protein that recruits USP7 to deubiquitinate ENO1, thereby protecting the ENO1 protein from degradation by the ERAD pathway (Fig. 5B). Moreover, consistent with our hypothesis, USP7 silencing reduced the expression of ENO1 at the protein level but had no effect on its transcriptional level (Fig. 5C and D).

We subsequently treated the cells with the protein synthesis inhibitor cycloheximide (CHX) and examined ENO1 protein levels at several time points, and it was clear that silencing either EMC2 or USP7 significantly shortened the half-life of the ENO1 protein (Fig. 5E/F & Fig. S4A/B).

Deng and Xu *et al.* reported that ENO1 is degraded via the ubiquitination pathway in gastric cancer and cholangiocarcinoma 29,30. To further determine whether ENO1 is also degraded in the same way in breast cancer, we treated MCF-7 and MDA-MB-231 cells with the ubiquitin-proteasome inhibitor MG-132. The results showed that MG-132 restored the expression levels of ENO1 in a time-dependent manner in both EMC2^ SH^ and USP7^ KD^ cell lines (Fig. 5G & Fig. S4C/D). In addition, we ruled out the possibility of ENO1 degradation via lysosomal or autophagic pathways, as neither the lysosomal inhibitor chloroquine (CQ) nor the autophagy inhibitor 3-methyladenine (3-MA) was able to restore ENO1 expression (Fig. 5G & Fig. S4C/D). Finally, we treated cells with MG-132 and confirmed that silencing EMC2 or USP7 significantly upregulated the ubiquitination level of ENO1 (Fig. 5H).

Collectively, the above results suggest that EMC2 protects ENO1 from ubiquitination-mediated degradation by recruiting USP7.

### ENO1 stabilizes the transcription factor B-MYB mRNA to initiate PDK1 transcription

**Figure 6:**
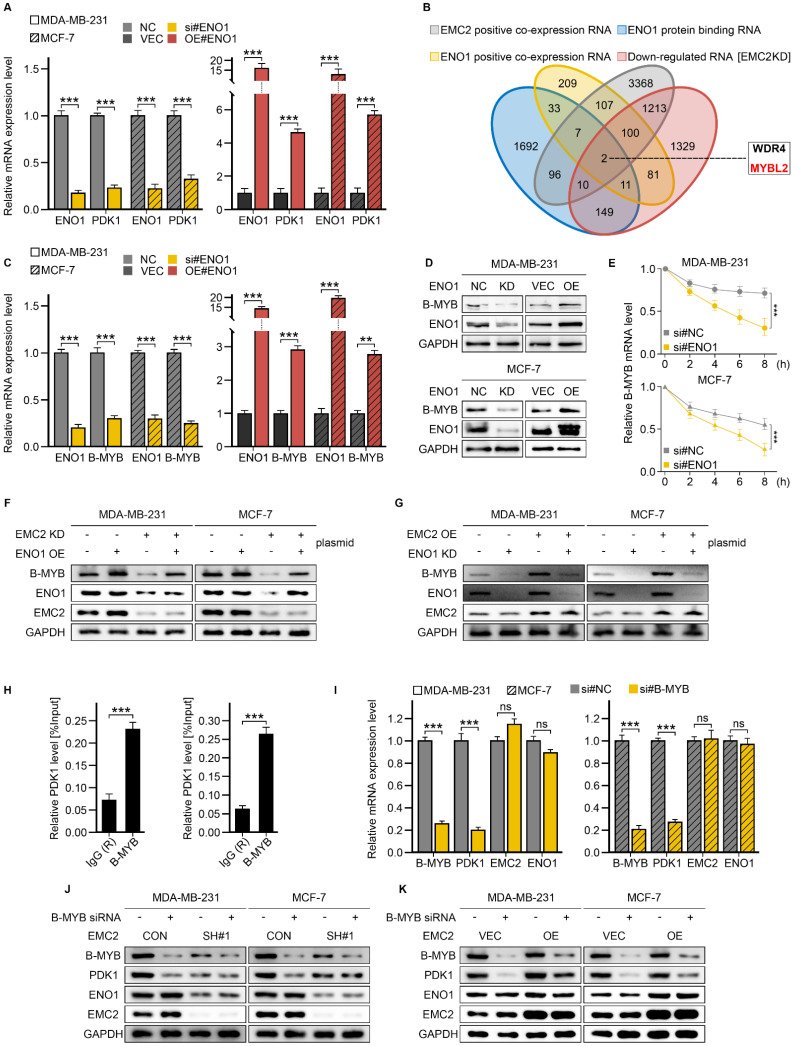
** ENO1-Mediated Regulation of B-MYB and PDK1. A.** qRT-PCR analysis of PDK1 expression following ENO1 knockdown or overexpression. **B.** Venn diagram analysis for identification of ENO1 target RNAs. **C.** qRT-PCR analysis of B-MYB expression following ENO1 knockdown or overexpression. **D.** Western blot analysis of B-MYB expression following ENO1 knockdown or overexpression. **E.** mRNA stability analysis using Actinomycin D (ActD, 5 μM) treatment at 0, 2, 4, 6, and 8 hours. **F-G.** Rescue experiments: Western blot analysis of B-MYB expression following: (F) ENO1 overexpression in EMC2-knockdown cells, (G) ENO1 knockdown in EMC2-overexpressing cells. **H.** ChIP-PCR analysis of B-MYB binding to PDK1 promoter region relative to IgG control. **I.** qRT-PCR analysis of PDK1, EMC2, and ENO1 expression following B-MYB knockdown. **J-K.** Western blot analysis of PDK1 and ENO1 expression following B-MYB knockdown.

Although RT‒qPCR revealed that ENO1 could upregulate PDK1 expression at the transcriptional level (Fig. 6A), our subsequent RIP-seq results revealed that ENO1 did not directly bind to PDK1 mRNA Table S5, and we chose to combine the RNA-seq and RIP-seq data to identify downstream targets of ENO1. Briefly, the candidate genes should be bound to ENO1 and demonstrate a positive correlation with the expression levels of both EMC2 and ENO1. As a result, we obtained two candidates, WD repeat domain 4 (WRD4) and MYB proto-oncogene like 2 (B-MYB) (Fig. 6B).

We then performed qRT‒PCR, western blot, and RNA stability experiments to further identify the target genes of ENO1, and it was clear that silencing ENO1 or EMC2 significantly downregulated the RNA and protein levels of B-MYB (Fig. 6C and D, Fig. S5A and B). Conversely, the opposite result was obtained (Fig. 6C and D, Fig. S5A and B). Moreover, the B-MYB mRNA half-life duration was positively correlated with the expression level of ENO1 or EMC2 (Fig. 6E, Fig. S5C). Furthermore, we did not observe similar effects of ENO1 or EMC2 on WDR4 Fig. S5D-I). We additionally performed rescue experiments, and the results suggested that the overexpression of ENO1 in EMC2^ KD^ cells could partially restore the expression of B-MYB (Fig. 6F). Similarly, silencing ENO1 suppressed the expression of B-MYB in EMC2OE cell lines (Fig. 6G). These findings suggest that EMC2 stabilizes downstream B-MYB mRNA levels by deubiquitinating ENO1 in breast cancer cells.

We next asked whether EMC2 activates the PDK1/AKT(T308)/mTOR(S2448) signaling pathway by upregulating B-MYB because B-MYB is a well-known transcription factor (31. We therefore performed ChIP‒qPCR in two cell lines. The primer pairs were designed on the basis of the B-MYB and PDK1 binding sequences predicted by the online transcription factor database JASPAR (Supplementary Table S5). The results demonstrated that the binding signals of B-MYB in the PDK1 promoter region were approximately 3-4-fold greater than those of the negative control IgG (Fig. 6H). Consistent with the ChIP‒qPCR results, the silencing of B-MYB significantly decreased the expression level of PDK1 (Fig. 6I‒K). In summary, rescue experiments revealed that the activation of the PDK1/AKT(T308)/mTOR(S2448) pathway caused by the overexpression of EMC2 was almost completely reversed by B-MYB silencing Fig. S5J and K).

In summary, our study demonstrated that EMC2 activates the PDK1/AKT(T308)/mTOR(S2448) signaling pathway by upregulating ENO1/B-MYB expression in breast cancer.

### EMC2 sensitizes tumor cells to PDK1/AKT inhibition

Given that EMC2 activates the PDK1/AKT/mTOR pathway in breast cancer, we hypothesized that EMC2 sensitizes tumor cells to PDK1/AKT inhibition. To test our hypothesis, we selected two drugs. BX-795 is a potent, selective PDK1 inhibitor that specifically inhibits the expression of PDK1 and its downstream target AKT (T308) (19,32. Capivasertib (AZD5363) is a novel pan-AKT inhibitor 33,34.

**Figure 7:**
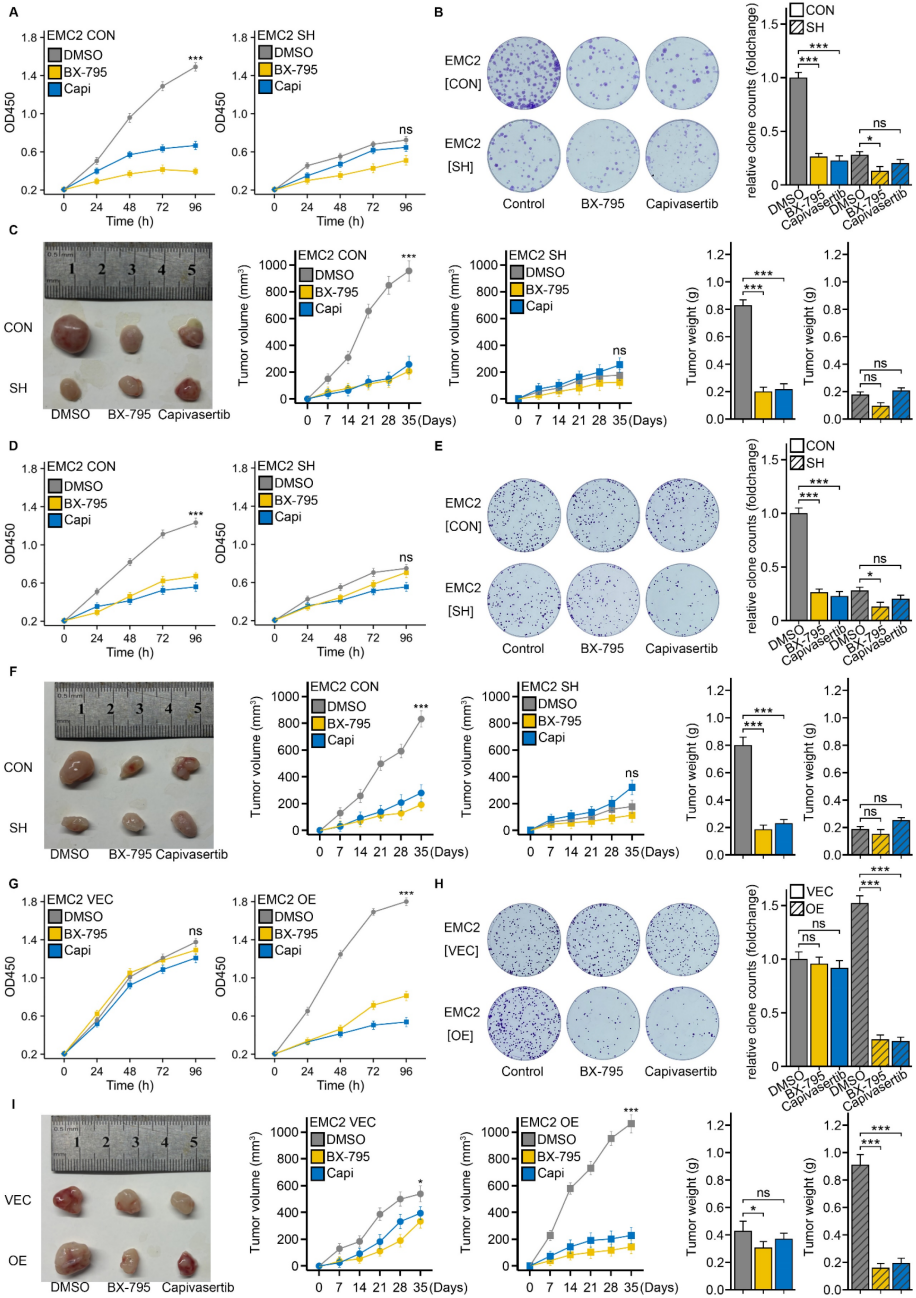
** EMC2 modulates breast cancer cell proliferation and tumor growth in response to AKT inhibition. A.** CCK-8 assay: EMC2 CON (control) and EMC2 SH (knockdown) cell lines (MDA-MB-231). **B.** Colony formation assay: EMC2 CON (control) and EMC2 SH (knockdown) cell lines (MDA-MB-231). **C.** Xenograft tumor experiments: EMC2 CON (control) and EMC2 SH (knockdown) cell lines (MDA-MB-231). **D-F.** Same as in A-C, but performed in MCF-7 cells (EMC2 CON vs EMC2 SH). **G-I.** Same as in A-C, but performed in SK-BR-3 cells (EMC2 VEC vs EMC2 OE).

First, we performed drug sensitivity experiments in MDA-MB-231 cells. CCK-8 and colony formation assays revealed that, compared with the control (DMSO), BX-795 or capivasertib significantly inhibited the proliferation of MDA-MB-231[EMC2 CON] cells *in vitro* (Fig. 7A and B). Concurrently, oral gavage of BX-795 or capivasertib markedly reduced the xenograft tumor growth rate and volume (Fig. 7C). Interestingly, when we performed the same experiments in EMC2-silenced MDA-MB-231[EMC2 SH] cells, the tumor cells became insensitive to both drugs, with no further reduction in proliferation caused by BX-795 or capivasertib *in vitro* or *in vivo* (Fig. 7A-C). We subsequently conducted the above experiments in MCF-7 cells and observed similar results, where the antitumor effects of BX-795 or capivasertib were abolished after EMC2 silencing (Fig. 7D-F).

To further confirm the association between EMC2 expression and drug efficacy, we chose the SK-BR-3 cell line, which has relatively low EMC2 expression, for rescue experiments. We then overexpressed EMC2 in SK-BR-3 cells and performed the aforementioned proliferation assays. Consistent with our hypothesis, at baseline, SK-BR-3 (EMC2 CON) cells were insensitive to either drug (Fig 7G-I). However, when we overexpressed EMC2 in SK-BR-3 cells, the proliferation of the experimental group (BX-795 or capivasertib) was significantly lower than that of the control group (DMSO) *in vitro* and *in vivo* (Fig 7G-I).

In summary, these results indicate that EMC2 sensitizes tumor cells to PDK1/AKT inhibition.

## Discussion

In this study, we demonstrated that EMC2 was aberrantly upregulated in breast cancer tissues, significantly enhanced the proliferation and metastasis potential of tumor cells, and increased the sensitivity of tumor cells to PDK1/AKT inhibition.

Mechanistically, EMC2 functions as a scaffold protein to recruit both USP7 and ENO1. Through the deubiquitinating activity of USP7, ENO1 avoids degradation by the ubiquitin-proteasome system. ENO1 stabilizes the oncogene B-MYB at the transcriptional level, which in turn activates the downstream PDK1/AKT(T308)/mTOR(S2448) signaling pathway, ultimately promoting tumorigenesis and progression.

Prior to our study, research investigating the mechanism of EMC2 in malignant cancer was lacking. Our study is the first to reveal that EMC2 functions as a scaffold protein in breast cancer. It recruits USP7 to deubiquitinate ENO1, thereby protecting ENO1 from ubiquitin-mediated degradation.

In conclusion, our study revealed that EMC2 drives breast cancer progression by activating the PDK1/AKT(T308)/mTOR(S2448) signaling pathway. Importantly, we pinpointed EMC2 as a potential target for PDK1/AKT inhibition. This crucial mechanism may facilitate the future development of targeted therapies.

The EMC is an abundant and highly conserved nine-subunit protein complex 35 located in the endoplasmic reticulum (ER). Research on EMC over the past decade has shown that disruption of its intact conformation broadly impacts the biosynthesis of cellular membrane proteins, further affecting cellular biological processes 36, including ER stress, viral infection 37, and lipid metabolic homeostasis 16. The role of EMC in membrane protein biosynthesis is largely attributed to its enzymatic activity in mediating the insertion of the TA protein into the lipid bilayer 38 or the cotranslational insertion of the N-terminal TMD 39.

Recent studies have provided new insights into the EMC complex. On the one hand, high-throughput proteomics has revealed a high degree of crosstalk 16 between the mammalian EMC complex and the ERAD pathway. On the other hand, depletion of EMC2 has been shown to affect the abundance of entire EMC complex members 40. However, it remains unclear whether EMC2 promotes tumor progression through ERAD-related mechanisms. Our research fills this gap in the field by demonstrating that EMC2 acts as a scaffold protein involved in regulating the ERAD degradation pathway *in vivo*.

ENO1, also known as 2-phospho-D-glycerate hydrolase, is a glycolytic enzyme 41. ENO1 is a typical multifunctional protein. In hepatocellular carcinoma (HCC), ENO1 can initiate a metastatic cascade 42 by activating the HGFR/WNT signaling pathway through phosphorylation. Moreover, by binding to 2-PG (2-phosphoglycerate) and regulating the pentose phosphate pathway (PPP), ENO1 can significantly increase tumorigenicity and platinum resistance in lung cancer 43. RNA-binding proteins (RBPs) are a class of proteins that can bind to and regulate the transcription or degradation of target RNAs 44. Recent studies have reported that ENO1, in addition to its metabolic activity, functions as an RBP to play a role in transcriptional regulation. For example, Sun *et al.* demonstrated that ENO1 activates the downstream PLCB1/HPGD signaling pathway by binding to the YAP1 mRNA translational element, ultimately leading to HCC progression 26. Similarly, Zhang *et al.* reported that ENO1 helps HCC cells escape ferroptosis 25 by degrading IRP1 mRNA. In gastric cancer, ENO1 promotes tumor growth 45 by stabilizing the expression of genes such as SOX9, VEGF-α, GPRC5A, and MCL-1. In this study, we revealed that ENO1 is protected from ubiquitin-mediated degradation by EMC2 29,30,46 and promotes tumor progression by stabilizing B-MYB mRNA.

B-MYB is a classic oncogenic transcription factor. Over the past few decades, the functions of B-MYB have been extensively elucidated by numerous studies. For example, B-MYB accelerates the cell cycle in colorectal cancer cells 47 by upregulating the E2F2/ERK/AKT signaling pathway, and suppressing IGFBP3 expression to promote proliferation and metastasis in non-small cell lung cancer 48. Our study revealed that B-MYB mRNA is stabilized by ENO1 in breast cancer and then initiates PDK1 transcription and ultimately activates the AKT (T308)/mTOR (S2448) signaling pathway.

BX-795 is a novel aminopyrimidine compound. Feldman first reported that it specifically inhibits PDK1 expression by competitively binding to the ATP active pocket. At low concentrations, BX-795 significantly reduces the expression of AKT (Thr308), without affecting AKT (Ser473) 49. Previously, BX-795 has shown multiple antitumor activities in malignancies such as neuroblastoma 32, liver cancer 50, prostate cancer 51, pancreatic cancer 18, and oral squamous cell carcinoma 52. Combining BX-795 can enhance the sensitivity of tumor cells to radiotherapy or chemotherapy.

Capivasertib (AZD5363), a novel pan-AKT inhibitor, is a widely noted antitumor drug. From its inception, it has shown broad-spectrum antitumor activity, especially in breast cancer 53. Capivasertib exhibits synergistic effects with both docetaxel and trastuzumab, which are commonly used chemotherapy or targeted therapy drugs for breast cancer 53. Notably, Capivasertib has already achieved favorable results in several clinical trials in breast cancer. The CAPItello-291 Phase III clinical trial reported that Capivasertib combined with fulvestrant significantly prolonged progression-free survival in HR-positive advanced breast cancer patients, some of whom had disease progression after CDK4/6 inhibitor treatment 54. For metastatic triple-negative breast cancer, the PAKT Phase III clinical trial reported that Capivasertib combined with paclitaxel extended the median progression-free survival of patients from 3.7 months to 9.3 months 55.

## Conclusion

In summary, our study provides strong evidence for the mechanism of EMC2 in the development and progression of breast cancer, and highlights its significant potential as a target for PDK1/AKT inhibition. These functions are mediated through its scaffold protein activity, which involves recruiting USP7 to deubiquitinate the RNA-binding protein ENO1, thus protecting ENO1 from ubiquitin-mediated degradation. As a result, the PDK1/AKT(T308)/mTOR(S2448) signaling pathway is activated through the transcription factor B-MYB, ultimately contributing to breast cancer progression.

Drug sensitivity experiments revealed that EMC2 overexpression sensitizes breast cancer cells to PDK1 or AKT inhibition both *in vitro* and *in vivo*, whereas EMC2 silencing has the opposite effect. These findings suggest that EMC2 is a potential therapeutic target for PDK1/AKT inhibition.

Furthermore, clinical analyses based on public databases indicate that EMC2 may also function as a biomarker for reduced overall survival in breast cancer patients. Our findings shed new light on the mechanisms driving breast cancer development and progression, and pave the way for future drug target screening endeavors.

However, this study has several limitations. We were unable to further construct a truncated ENO1 protein to clarify the binding site of the USP7-ENO1 complex. Nevertheless, we believe that this does not impact the main conclusions of the study. Moreover, in future work, we intend to apply BX-795 and capivasertib to organoid or PDX models to further corroborate the conclusion that EMC2 sensitizes tumor cells to PDK1/AKT inhibition therapy, serving as a complement to existing *in vivo* and *in vitro* drug sensitivity experiments.

## Supplementary Material

Supplementary figures.

Supplementary table 1.

Supplementary table 2.

Supplementary table 3.

Supplementary Table 4 (anti EMC2 IP-MS results) & Supplementary Table 5 (anti ENO1 RIP-seq results).

## Abbreviations

EMC2: Endoplasmic reticulum membrane protein complex subunit 2
PDK1: Pyruvate dehydrogenase kinase isozyme 1
AKT: Protein kinase B (PKB)
ENO1: Enolase 1
HR: Hormone receptor
ER: Endoplasmic reticulum/Estrogen receptor
TA: Tail-anchored (protein)
TMD: Transmembrane domain
ERAD: Endoplasmic reticulum-associated protein degradation
OS: Overall survival
RFS: Recurrence-free survival
CCK-8: Cell counting kit-8
CDK1: Cyclin-dependent kinase 1
EMT: Epithelial-mesenchymal transition
PI3K: Phosphatidylinositol 3-kinase
mTOR: Mammalian target of rapamycin
USP7: Ubiquitin specific peptidase 7
DUB: Deubiquitinating enzyme
WRD4: WD repeat domain 4
HCC: Hepatocellular carcinoma
PPP: Pentose phosphate pathway
GSEA: Gene set enrichment analysis
RNA-seq: RNA sequencing
RIP-seq: RNA immunoprecipitation sequencing
IP-MS: Immunoprecipitation-mass spectrometry
ChIP-qPCR: Chromatin immunoprecipitation-quantitative PCR
qRT-PCR: Quantitative real-time reverse transcription PCR
DMSO: Dimethyl sulfoxide
IC50: Half-maximal inhibitory concentration
FBS: Fetal bovine serum
ATCC: American type culture collection
STR: Short tandem repeat
CQ: Chloroquine
PFA: Paraformaldehyde
DAB: Diaminobenzidine
HE: Hematoxylin and eosin
TCGA: The cancer genome atlas
NC: Nitrocellulose
