# Supplementary material for: EMC2 promotes breast cancer progression and enhances sensitivity to PDK1/AKT inhibition by deubiquitinating ENO1
Source: Int J Biol Sci. 2025 Mar 24;21(6):2629–46. doi: 10.7150/ijbs.109192 (PMC12035906; doi:10.7150/ijbs.109192)
Supplement: Supplementary file 1 — Supplementary figures. [file ijbsv21p2629s1.pdf]

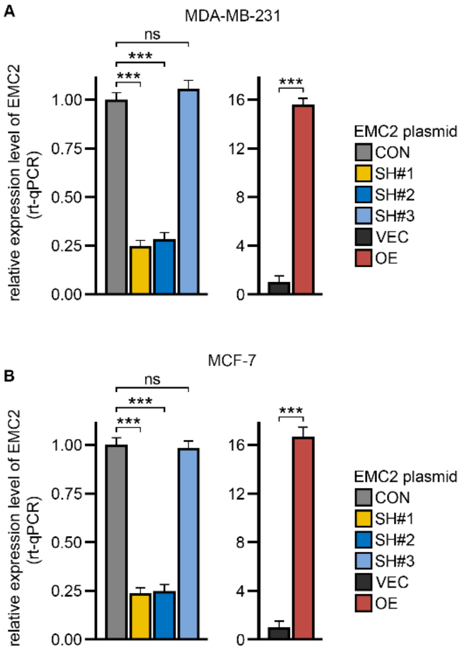

Figure S1. Validation of EMC2 Gene Manipulation

A-B. Quantitative RT-PCR validation of EMC2 mRNA expression following plasmid-mediated knockdown or overexpression.

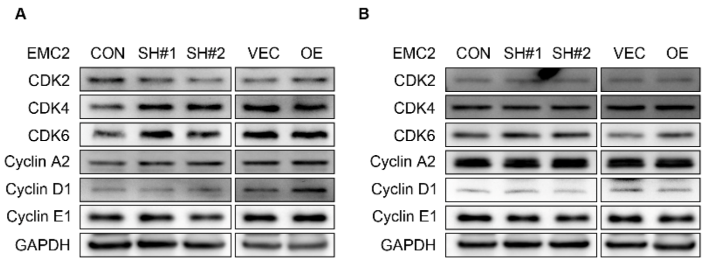

Figure S2. Additional Cell Cycle Analysis

A-B. Western blot analysis of cell cycle checkpoint proteins.

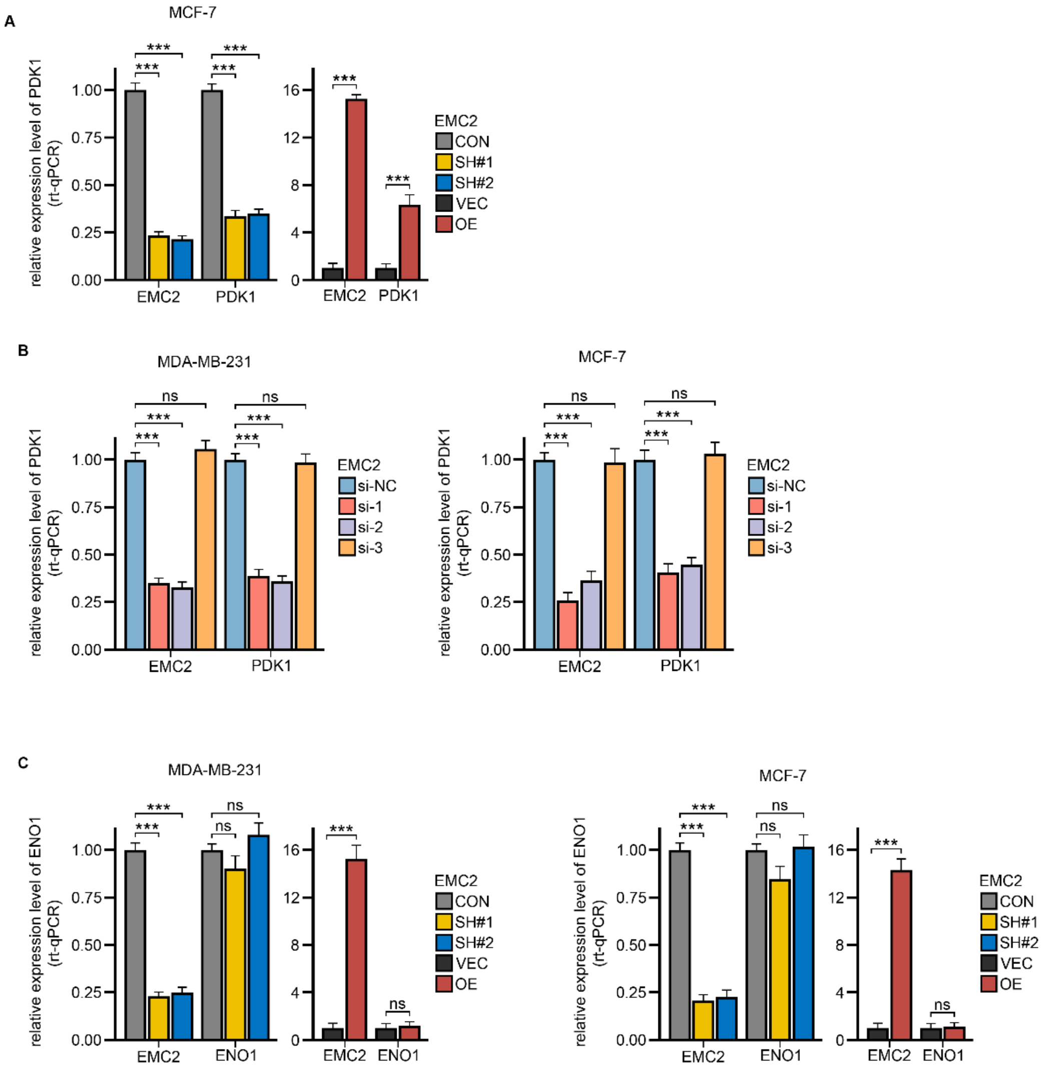

Figure S3. Additional Expression Analysis

A. qRT-PCR analysis of PDK1 expression following EMC2 knockdown or overexpression.

B. qRT-PCR analysis of PDK1 expression following transient EMC2 knockdown using siRNA.

C. qRT-PCR analysis of ENO1 expression following EMC2 knockdown or overexpression.

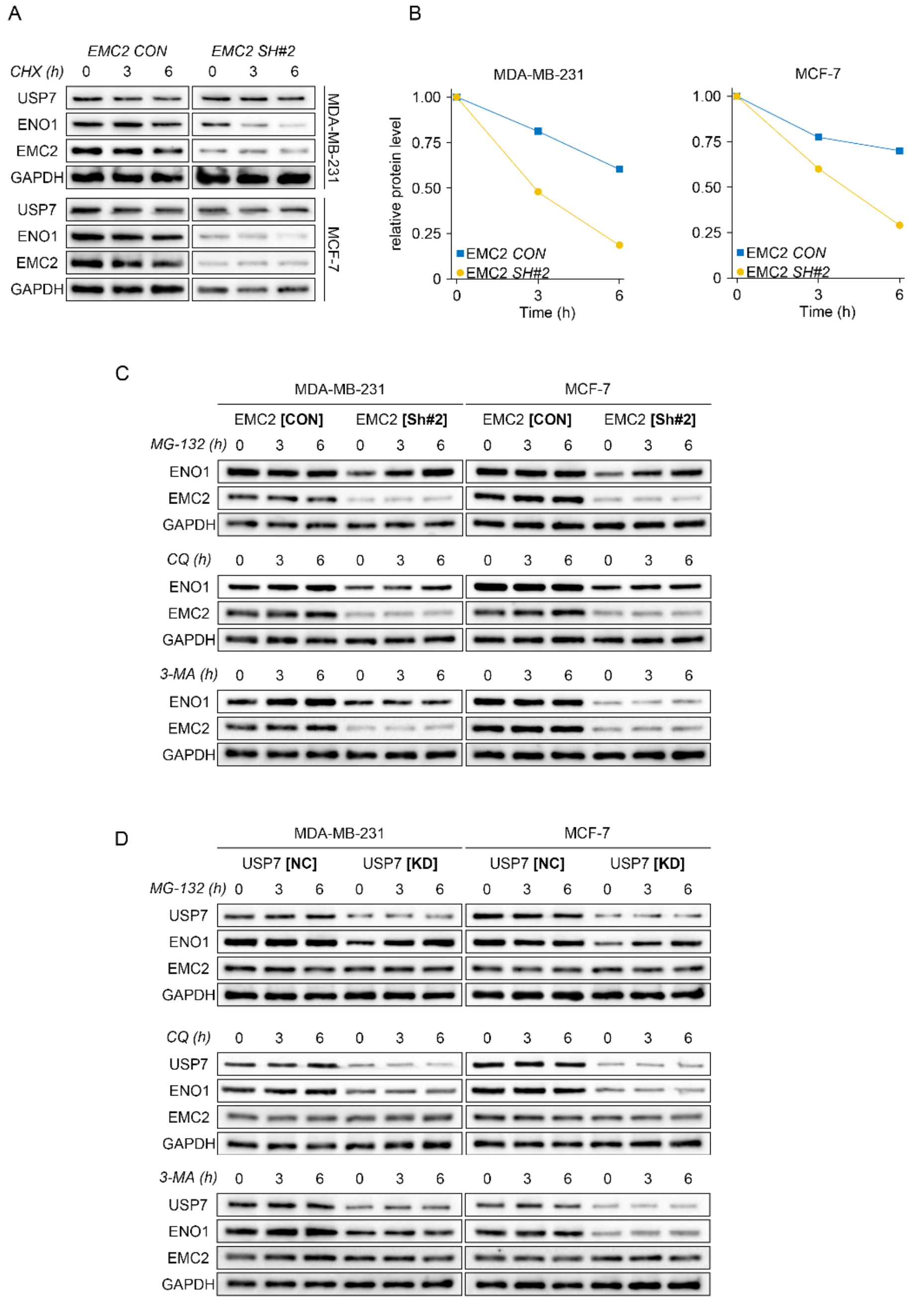

Figure S4. ENO1 protein Half-life assay and Degradation inhibit assay

A-B. Protein stability analysis using cycloheximide chase assay (CHX, 50 μg/mL) at 0, 3, and 6 hours.

C-D. ENO1 protein levels following treatment with protein degradation inhibitors:

- MG-132 (proteasome inhibitor, 10 μM)
- Chloroquine (CQ, lysosomal inhibitor, 50 μM)
- 3-Methyladenine (3-MA, autophagy inhibitor, 10 mM)

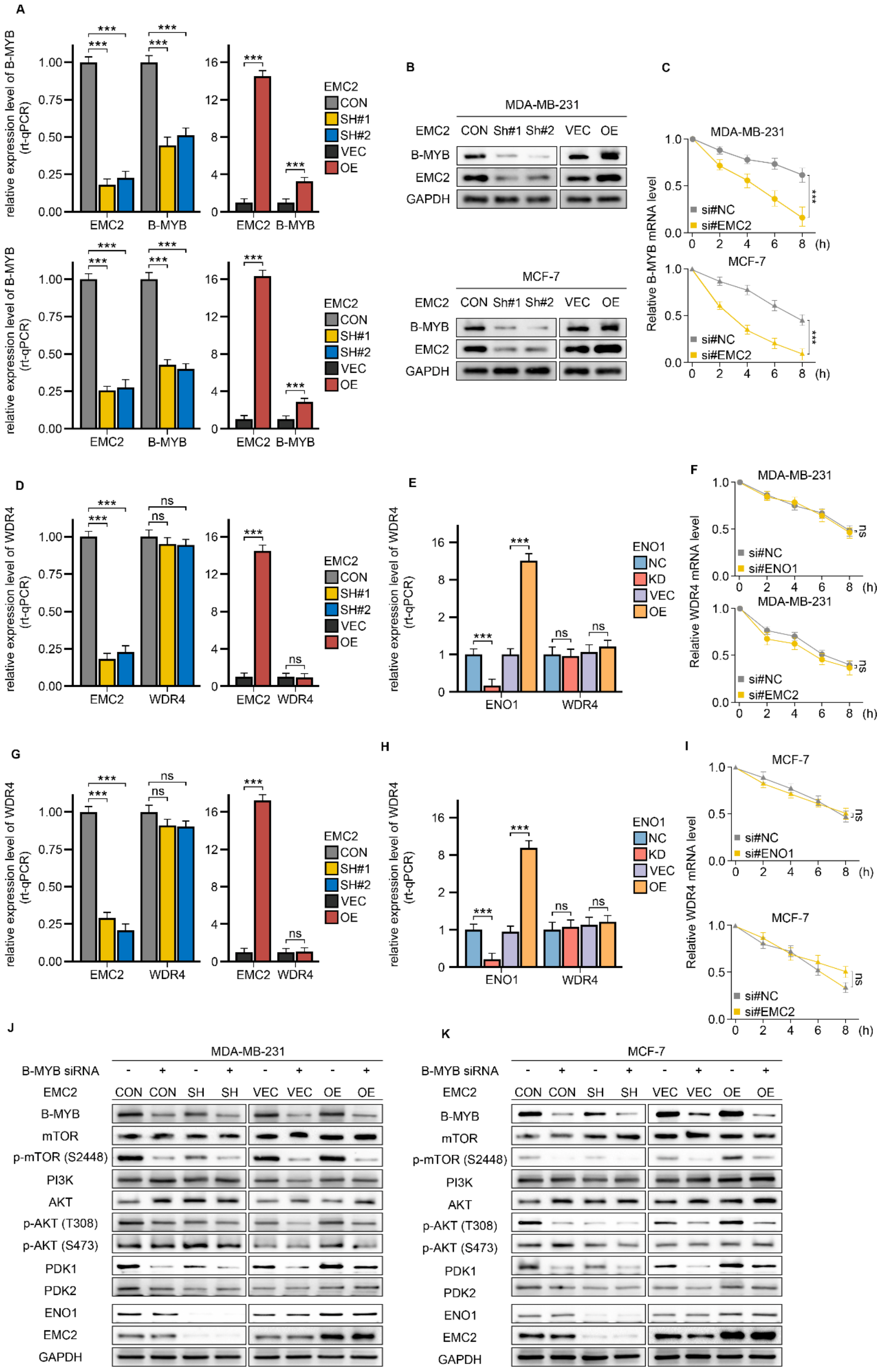
A
relative expression level of B-MYB (rt-qPCR)
EMC2
CON
SH#1
SH#2
VEC
OE
EMC2 B-MYB
***
B
MDA-MB-231
EMC2 CON Sh#1 Sh#2 VEC OE
B-MYB
EMC2
GAPDH
MCF-7
C
MDA-MB-231
MCF-7
Relative B-MYB mRNA level
si#NC
si#EMC2
(h)
D
relative expression level of WDR4 (rt-qPCR)
EMC2 WDR4
ns
E
ENO1
NC
KD
VEC
OE
ENO1 WDR4
F
Relative WDR4 mRNA level
si#ENO1
G
H
I
J
MDA-MB-231
B-MYB siRNA
EMC2 CON CON SH SH VEC VEC OE OE
B-MYB
mTOR
p-mTOR (S2448)
PI3K
AKT
p-AKT (T308)
p-AKT (S473)
PDK1
PDK2
ENO1
EMC2
GAPDH
K
MCF-7

Figure S5. ENO1-Mediated Regulation of B-MYB and PDK1

A. B-MYB expression following EMC2 knockdown or overexpression (qRT-PCR).

B. B-MYB expression following EMC2 knockdown or overexpression (Western blot).

C. B-MYB mRNA stability analysis after treating with Actinomycin D (ActD, 5 μM) at 0, 2, 4, 6, and 8 hours.

D. WDR4 expression following EMC2 knockdown or overexpression (qRT-PCR; MDA-MB-231).

E. WDR4 expression following ENO1 knockdown or overexpression (qRT-PCR; MDA-MB-231).

F. WDR4 mRNA stability analysis after treating with Actinomycin D (ActD, 5 μM) at 0, 2, 4, 6, and 8 hours (MDA-MB-231).

G. WDR4 expression following EMC2 knockdown or overexpression (qRT-PCR; MCF-7).

H. WDR4 expression following ENO1 knockdown or overexpression (qRT-PCR; MCF-7).

I. WDR4 mRNA stability analysis after treating with Actinomycin D (ActD, 5 μM) at 0, 2, 4, 6, and 8 hours (MCF-7).

J. PDK1 and ENO1 expression following B-MYB knockdown (Western blot; MDA-MB-231).

K. PDK1 and ENO1 expression following B-MYB knockdown (Western blot; MCF-7).
