# Supplementary material for: EMC2 promotes breast cancer progression and enhances sensitivity to PDK1/AKT inhibition by deubiquitinating ENO1
Source: Int J Biol Sci. 2025 Mar 24;21(6):2629–46. doi: 10.7150/ijbs.109192 (PMC12035906; doi:10.7150/ijbs.109192)
Supplement: Supplementary file 2 — Supplementary table 1. [file ijbsv21p2629s2.pdf]

Supplementary Table 1

Sequence of siRNA or shRNA used in this research

Sequence used for knockdown plasmid construction

sh-EMC2#1	GTGGAAGTTGGAGAAGAATTA
sh-EMC2#2	AGGCATGAGATTTGAAGCCAT
sh-EMC2#3	CAGGTGATGATTGCAGCACTA
sh-ENO1	CCGGCGTGAACGAGAAGTCCTGCAACTCGAGTTGCAGGACTTCTCGTTCACGTTTTTG
sh-CON	TTCTCCGAACGTGTCACGT

Sequence used for overexpression plasmid construction

OE-EMC2	NM_014673 (CDS region)
OE-ENO1	NM_001428.5 (CDS region)
OE-VEC	Empty vector

siRNA sequense

si-ENO1#1	CGUACCGCUUCCUUAGAACUUdTdT (sense)<br>AAGUUCUAAGGAAGCGGUACGdTdT (antisense)
si-ENO1#2	GAAUGUCAUCAAGGAGAAAUAdTdT (sense)<br>UAUUUCUCCUUGAUGACAUUCdTdT (antisense)
si-ENO1#3	CCACUGUUGAGGUUGAUCUCUdTdT (sense)<br>AGAGAUCAACCUCAACAGUGGdTdT (antisense)
si-MYBL2#1	CCGUCCCUCCUACCAUAAAdTdT (sense)<br>UUUAUGGUAGGAGGGACGGdAdG (antisense)
si-MYBL2#2	CCAAGAGCACACCUGUUAAdTdT (sense)<br>UUAACAGGUGUGCUCUUGGdGdC (antisense)
si-MYBL2#3	CCUCCCAGUUUCUGAACUUdTdT (sense)<br>AAGUUCAGAAACUGGGAGGdGdC (antisense)
si-USP7#1	GAGCGACCTTACCCAAGTT (sense)<br>AACUUGGGUAAGGUCGCUC (antisense)
si-USP7#2	UAAGGACCCUGCAAAUUAU (sense)<br>AUAAUUUGCAGGGUCCUUA (antisense)
